# Changes in blood hemoglobin and blood gases PaO_2_ and PaCO_2_ in severe COPD overa three-year telemonitored program of long-term oxygen treatment

**DOI:** 10.1186/2049-6958-7-15

**Published:** 2012-07-17

**Authors:** Roberto W Dal Negro, Silvia Tognella, Luca Bonadiman, Paola Turco

**Affiliations:** 1 Department of Respiratory Diseases, ULSS22 Veneto Region, Orlandi Hospital, Via Ospedale 2, 37012, Bussolengo, VR, Italy; 2 National Centre of Studies of Pharmacoeconomics and Respiratory pharmacoepidemiology (CESFAR), Via Gabriele Rossetti 4, Bussolengo, Verona, Italy

**Keywords:** Anemia, COPD, Hemoglobin, LTOT, Polyglobulia, Telemedicine

## Abstract

**Background:**

Information on the effects of long-term oxygen treatment (LTOT) on blood hemoglobin (Hb) in severe COPD are limited. The aim was to assess blood Hb values in severe COPD, and investigate the time-course of both Hb and blood gas changes during a 3-year telemetric LTOT.

**Methods:**

A cohort of 132 severe COPD patients (94 males; 71.4 years ± 8.8 sd), newly admitted to the tele-LTOT program, was investigated. Subjects were divided according to their original blood Hb: group A: <13 g/dL; group B: ≥13 < 15 g/dL; group C: ≥ 5 < 16 g/dL; group D: ≥16 g/dL. Blood Hb (g/dL), PaO_2_ and PaCO_2_ (mmHg), SaO_2_ (%), and BMI were measured at LTOT admission (t_0_), and at least quarterly over three years (t_1_-t_3_). Wilcoxon test was used to compare t_0_ vs. t_1_ values; linear regression to assess a possible Hb-BMI relationship; ANOVA to compare changes in Hb time-courses over the 3 years.

**Results:**

LTOT induced a systematic increase of PaO_2_, and changes were significant since the first year (from 52.1 mmHg ± 6.6sd to 65.1 mmHg ± 8.7 sd, p < 0.001). Changes in SaO_2_ were quite similar. Comparable and equally significant trends were seen in all subgroups (p < 0.001). PaCO_2_ dropped within the first year of LTOT (from 49.4 mmHg ± 9.1sd to 45.9 mmHg ±7.5 sd, p < 0.001): the t_0_-t_1_ comparison proved significant (p < 0.01) only in subgroups with the highest basal Hb, who showed a further PaCO_2_ decline over the remaining two years (p < 0.001). Hb tended to normalization during LTOT only in subgroups with basal Hb > 15 g/dl (ANOVA p < 0.001); anemic subjects (Hb < 13 g/dl) ameliorated not significantly in the same period (ANOVA = 0.5). Survival was independent of the original blood Hb. Anemia and polyglobulia are differently prevalent in COPD, the latter being the most represented in our cohort. LTOT affected both conditions, but to a different extent and according to different time-courses. The most striking Hb improvement was in polyglobulic patients in whom also PaO_2_, PaCO_2_ and SaO_2_ dramatically improved. In anemic subjects effects were smaller and slower, oxygenation being equally ameliorated by LTOT.

**Conclusions:**

LTOT effects on Hb and PaCO_2_ are regulated by an Hb-dependent gradient which seems independent of the original impairment of blood gases and of effects on oxygenation.

## Background

Chronic obstructive pulmonary disease (COPD) is currently defined as characterised by airflow limitation which is not fully reversible and able to produce systemic consequences (Global Initiative for Chronic Obstructive Lung Disease [1]).

Besides its effect on lung function, the chronic impairment of the respiratory system due to COPD has long-term multi-organ consequences on the bone and liver metabolism, heart and cardiovascular system, brain, kidney, and skeletal muscles.

Many chronic diseases have been shown to affect hematopoiesis, and also COPD may be associated to this kind of disorder which represents further aspects of the multiple involvement of the disease.

Even though COPD had in the past been commonly regarded as a determinant of polyglobulia rather than of anemia [2], it is currently thought that the latter condition occurs more frequently than expected (in 10-15% of severe COPD patients) [3], and it is presumed to be associated with ongoing inflammation, altered erythropoietin function, and poor marrow production [4].

There is still limited information concerning the distribution of blood hemoglobin (Hb) concentrations in the COPD population [3,5,6], although both the direct contribution of anemia in causing breathlessness and the tendency to a secondary polyglobulia have long been accepted in severe COPD [2]. On the other hand, limited information is available concerning the effects of long-term domiciliary oxygen treatment on Hb and concomitantly on blood gases in these circumstances [7].

The present study aimed to assess the distribution of blood Hb values in a population of severe COPD patients with chronic respiratory failure, and to investigate the time course of both Hb and blood gas changes during a 3-year program of telmonitored long-term oxygen therapy (LTOT).

## Methods

A cohort of 132 consecutive non-smoker (87.1% former smoker), severe COPD patients was studied (94 males; mean age 71.4 years ± 8.8 sd). All patients had been admitted as first-time candidates to the domiciliary LTOT program over the last three years (2008-2010) and were selected from the dedicated institutional database (ISO 9001-2000 certified since 1999) [8]. In order to avoid possible bias in the study, both the selection procedures and the subsequent creation and management of the data bank were outsourced to IT professionals employed by a third party, i.e. not working in the Lung Department. All COPD patients had a requirement of LTOT according to our regional guideline; they were managed according to daily telemonitoring of vital signs (such as heart rate, SAP, DAP, oxygen saturation (SaO_2_), oxygen consumption) and compliance to oxygen therapy, by a program which has been operating in our Lung Department for a a number of years (Contel System, Air Liquide – VitalAire SA, Paris, France) [9]. From the point of view of their basic pharmacological respiratory treatment (directly delivered by the Lung Department on a regular basis), 96.2% of patients (127/132) were daily assuming long-acting β_2_-adrenergics (LABA) and long-acting anti-cholinergics (LAMA), and 91.7% (121/132) were also assuming inhaled corticosteroids (ICS). All patients were also using diuretics (furosemide) and were equally supplemented with vitamins and other nutritional supports (essential amino acids) [10]. Their adherence to both oxygen (an average consumption of 2.3 L/min liquid oxygen ± 0.8 sd, for at least 17 hours/day; range 0.5-3.0 L/min) and pharmacological treatments was assessed monthly by professional caregivers, and defined as very good (>75% of the prescribed doses in all subjects). The mean% patients’ survival at three years and the mean prevalence/patient of comorbidities (e.g. due to cardiovascular, metabolic, renal, gastrointestinal and neurological causes) were also measured.

Blood Hb (g/dL), PaO_2_ and PaCO_2_ (mm Hg), SaO_2_ (%), and BMI were measured in each patient at admission to the telmonitored LTOT program (t_0_), and at least three times/year over the following three years (t_1_-t_3_). Each variable was expressed yearly as mean ± sd of all measurements performed in the period.

The whole cohort was then divided into four subgroups, according to the subjects’ original blood Hb: group A: < 13 g/dL; group B: ≥ 13 < 15 g/dL; group C: ≥ 15 < 16 g/dL; group D: ≥ 16 g/dL.

For statistical analysis the Wilcoxon test was used to compare t_0_ vs. t_1_ values of all variables, linear regression was used to compare Hb and BMI values, and ANOVA to compare changes in Hb time courses of the entire sample and of each subgroup over the 3-year period; p < 0.05 was assumed as the minimum level of significance.

## Results

Basal lung function, blood gases, and Hb measured in the whole population are reported in Table 1, while the distribution of patients within the four subgroups together with their corresponding mean blood Hb values are reported in Table 2. Mean basal Hb level was lower in females by 0.8 g/dL, and this difference was maintained during the entire survey (p = ns). In the entire cohort, 18.2% of patients (24/132; 15 males) had a mean BMI < 23 kg/m^2^, while clearly pathological Hb levels were found in only 37.1% of patients (49/132), anemic subjects being less numerous than polyglobulic subjects (11.3% vs. 25.8%, respectively). No significant gender differences were found.

**Table 1:** Mean basal values ± sd for lung function, blood gases, Hb and BMI (n = 132) in the study group

	**Whole sample**	**Males**	**Females**
	**(n = 132)**	**(n = 94)**	**(n = 38)**
FEV_1_% pred.	35.3 ± 11.8	36.6 ± 16.4	35.7 ± 14.1
FVC% pred.	60.2 ± 10.7	59.2 ± 13.5	62.5 ± 19.3
FEV_1_/FVC%	58.3 ± 8.1	61.8 ± 7.9	57.1 ± 8.6
pH	7.41 ± 0.05	7.40 ± 0.05	7.41 ± 0.04
PaO_2_ mm Hg	52.1 ± 6.6	52.2 ± 6.5	51.8 ± 7.1
PaCO_2_ mm Hg	49.4 ± 9.1	48.9 ± 8.9	50.5 ± 9.5
HCO_3_ mmol/L	30.5 ± 4.5	30.0 ± 3.9	31.4 ± 5.5
SaO_2_%	86.3 ± 5.7	86.5 ± 5.7	85.8 ± 6.0
Hb g/dL	15.1 ± 1.9	15.4 ± 2.0	14.5 ± 1.8
BMI	27.4 ± 6.8	27.8 ± 6.9	26.5 ± 6.8

BMI, body mass index; FEV_1_, forced expiratory volume in 1 second; FVC, forced vital capacity; Hb, Hemoglobin; PaCO_2_, partial pressure of arterial carbon dioxide; PaO_2_, partial pressure of arterial oxygen; SaO_2_, oxyhemoglobin saturation.

**Table 2:** Distribution of subjects in the four subgroups according to their basal hemoglobin (Hb) values, and corresponding mean Hb values ± sd

**Subgroups**	**n (%)**	**Hb (mean ± sd)**
A (< 13 g/dL)	15/132 (11.3%)	11.7 g/dL ± 0.9
B (≥ 13 <15 g/dL)	42/132 (31.8%)	14.1 g/dL ± 0.5
C (≥ 15 <16 g/dL)	41/132 (31.1%)	15.4 g/dL ± 0.3
D (≥ 16 g/dL)	34/132 (25.8%)	17.5 g/dL ± 1.3

**Figure 1:**
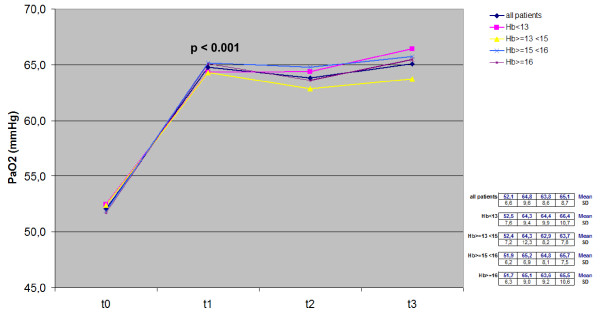
**Time course of PaO**_**2**_**values in the whole cohort and in each subgroup:*t*test between t**_**0**_**and t**_**1**_**.** Hb, hemoglobin; PaO_2_, partial pressure of arterial oxygen.

**Figure 2:**
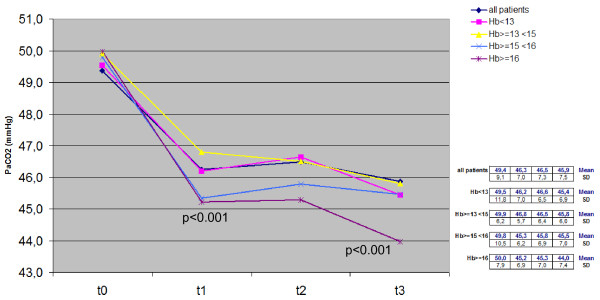
**Time course of PaCO**_**2**_**values in the whole cohort and in each subgroup:*t*test between t**_**0**_**and t**_**1**_**.** Hb, hemoglobin; PaCO_2_, partial pressure of arterial carbon dioxide.

**Figure 3:**
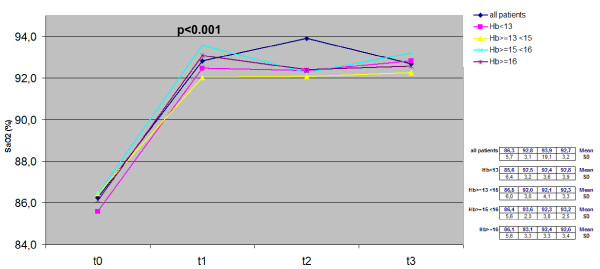
**Time course of SaO**_**2**_**values in the whole cohort and in each subgroup:*t*test between t**_**0**_**and t**_**1**_**.** Hb, hemoglobin; SaO_2_, oxyhemoglobin saturation.

**Figure 4:**
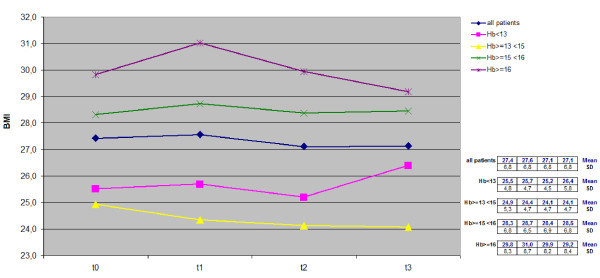
**Time course of BMI values in the whole cohort and in each subgroup.** BMI, body mass index; Hb, haemoglobin.

**Figure 5:**
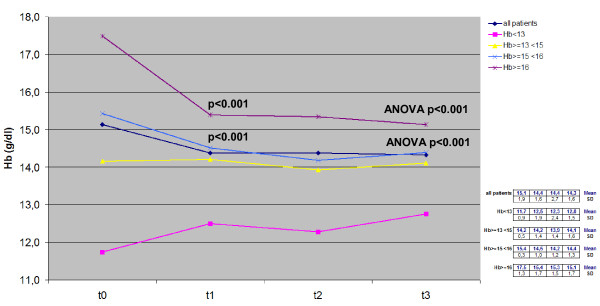
**Time course of Hb values in the whole cohort and in each subgroup:*t*test between t**_**0**_**and t**_**1,**_**and anova over time.** Hb, haemoglobin.

Trends for PaO_2_, PaCO_2_, SaO_2_, BMI and Hb measured over the 3-year period in the whole cohort and in the four subgroups with different basal Hb levels are reported in Figures 1, 2, 3, 4, 5.

LTOT induced a systematic increase of arterial PaO_2_ in all patients, and changes proved highly significant already within the first year of LTOT (from 52.1 mm Hg ± 6.6 sd to 65.1 mm Hg ± 8.7 sd, p < 0.001); then PaO_2_ values remained unchanged over the remaining two years. The corresponding trends in each subgroup were comparable to that of the whole cohort, and the comparison between t_0_ and t_1_ mean values was equally statistically significant in all subgroups (p < 0.001) (Figure 1). A quite similar time course was observed for SaO_2_ (Figure 2).

Also the great proportion of changes of arterial PaCO_2_ measured in the entire cohort of patients occurred within their first year of LTOT (from 49.4 mm Hg ± 9.1 sd to 45.9 mm Hg ± 7.5 sd, p < 0.001). In this case, the corresponding PaCO_2_ trends in each subgroup were very similar to that of the entire cohort, but the comparison between t_0_ and t_1_ mean values proved statistically significant only in group C and D, such as in those with the highest original Hb values (from 49.8 mm Hg ± 10.5 sd to 45.3 mm Hg ± 6.2 sd; p < 0.05, and from 49.9 ± 7.9 sd to 45.2 mmHg ± 6.9 sd; p < 0.01, respectively). Moreover, subgroup D (i.e. subjects with basal Hb > 16 g/dL) showed the highest drop in arterial PaCO_2_ at t_1_, and also showed a further progressive PaCO_2_ decline towards normal values over the remaining two years of LTOT (p < 0.001) (Figure 3).

BMI proved significantly related to Hb values in basal condition (p < 0.03; r = 0.23). The general trends of BMI for the whole population and the four different subgroups persisted without any significant change over the total 3-year control period (Figure 4).

All changes measured in Hb values are summarized in Figure 5. Differently from the time course (absolutely flat) registered in the whole cohort, peculiar trends were measured in the four subgroups. In particular, Hb values tended to drop (i.e. to normalize) since the first year of LTOT in those subgroups originally characterized by the highest mean Hb values (i.e. > 15 g/dL) (ANOVA p < 0.001). On the other hand, the anemic subgroup (Hb < 13 g/dL) showed a progressive but not significant (ANOVA = 0.5) increase of Hb mean values over the 3-year period; the highest change occurred within the first year also in these subjects even though the variation never reached statistical significance (p < 0.08).

Finally, the overall survival was 73.4% at three years: 73.3% in group A; 71.4% in group B; 73.2% in group C, and 76.5% in group D (p = ns), while the overall prevalence of comorbidities was 2.0/patient in the cohort: 2.2/p in group A; 1.9/p in group B; 2.0/p in group C, and 2.1/p in group D, respectively.

## Discussion

Lung function and blood hemoglobin have been investigated extensively in COPD, and continuous long-term oxygen treatment (>15 hours/day) is generally regarded as of significant benefit in hypoxemic patients suffering from COPD [11].

Nevertheless, the true mechanisms underlying the improved clinical outcomes (e.g. hospitalization rate, QoL, or survival) during LTOT still have to be clarified, although it is well known since long ago that long-term oxygen decreases hematocrit values, pulmonary vascular resistances, and improves brain performance [12,13].

Anemia and polyglobulia are conditions differently represented in COPD, the latter being the most prevalent in our cohort. Several mechanisms have been suggested to explain the former condition, such as the shortening of red blood cell lifespan and sequestration of iron in macrophages, which can lead to the so-called “anemia of chronic disease” (ACD) [14,15]. ACD represents a further aspect of the multiple involvement of COPD and is presently regarded as an immune driven abnormality that occurs in many inflammatory diseases [3]. It is a condition which is associated with increased morbidity and mortality in chronic renal failure [16,17], congestive heart failure [18-20], HIV and hepatitis C infection [21-23], digestive disease [3,24], and cancer [25]. Furthermore, anemia is also associated with disability, impaired physical performance, lower muscle strength in individuals over 65 yrs of age [6], and reduced health-related quality of life (HRQL). Recent reports suggest that anemia is highly prevalent in patients with COPD and associated with increased mortality [6,26].

Polyglobulia had been traditionally accepted as a secondary event due to chronic hypoxemia occurring in a great proportion of COPD patients [2,27]; nevertheless, it has been supposed that the normal values found in the majority of COPD patients are the result of a balance between the trend towards a decreased red cell mass and an opposite trend towards an increased red cell mass due to the erythropoietic effect of erythropoietin in these subjects [28].

Concerning the prevalence of these two conditions, the anemic condition was found in 11.3% while polyglobulia was found in 25.8% of patients of the present study, and these findings are in good agreement with the current literature [3,26,29,30].

LTOT proved to affect both conditions in our cohort, but to at different extent and according to different time courses. In particular, the most striking changes in Hb values were obtained in polyglobulic patients in whom >90% of the effect (i.e. a decrease) was seen within the first year of LTOT. In these patients, also PaO_2_, PaCO_2_ and SaO_2_ dramatically improved in the same period; only PaCO_2_ showed a further, progressive decrease compared to the original Hb values over the remaining two years of the survey.

On the contrary, the effect of LTOT on anemic subjects was smaller and much slower. Actually, these patients were originally characterized by a lower BMI (25.5 vs. 28.3 and 29.8 kg/m^2^ of patients with Hb values >15 g/dL), and required a longer period (a further couple of years) for an improvement to be seen in both their Hb and BMI, thus emphasizing the crucial role of metabolic and nutritional aspects. On the other hand, the prognostic value of an improved nutritional status has been demonstrated in severe COPD to be mainly related to the increase in BMI and other related indices [31]: BMI values proved directly related to Hb values in our population during LTOT, particularly in the anemic subgroup during the third year of the survey (Figures 4 and 5), thus emphasizing the possible role of LTOT in ameliorating the nutritional status (Hb included) of anemic subjects.

Moreover, even though oxygenation resulted equally improved by LTOT in polyglobulic and anaemic patients, the latter subset of subjects obtained a smaller and delayed benefit from LTOT in terms of PaCO_2_ amelioration, likely due to their original lower Hb content in blood. In other words, the present data suggest the presence of an Hb-dependent gradient in the LTOT effect on Hb, which appears to be independent of the extent of the original impairment of blood gases.

These data tend to confirm those from a previous study carried out on 13 non selected subjects in whom no benefits were obtained in terms of changes in Hb and hematocrit in COPD patients admitted to LTOT for two years, even though their QoL and both anxiety and depression scores improved significantly [32].

The present data are not in agreement with the evidence of poorer outcomes and survival at three years in COPD patients with anemia (hematocrit <35%) when compared to those with polyglobulia (24% vs. 70%, respectively) [26]. In particular, in our cohort of 132 severe COPD patients on LTOT, mortality and the prevalence of comorbidities resulted equally distributed in polyglobulic and anemic subjects at the end of the 3-year survey (26.7% vs. 23.5%, and 2.2/patient vs. 2.1/patient, respectively).

## Conclusions

Differently from earlier studies [32], the data of our survey emphasize the good results of a strict domiciliary telmonitored protocol for LTOT: Hb values tended to improve substantially in parallel to blood gases already from the first year of LTOT in the great majority of severe COPD subjects. Actually, better results were mostly achieved in polyglobulic subjects who, on the other hand, represented the most frequent picture of severe COPD in our cohort. As suggested in previous studies [26;29], these patients proved more sensitive to the effects of long-term oxygen than anemic patients who required a longer period of LTOT to obtain smaller results. While it is plausible that in severe COPD the anemic condition may represent an independent factor that can affect the extent and time course of long-term metabolic outcomes during LTOT, it is however confirmed that LTOT plays a crucial role in optimizing blood gases independently of Hb.

## Competing interests

The authors declare that they have no competing interests.
